# Supplementary material for: Characterization and Bioactivity of Piper chaudocanum L. Extract-Doped ZnO Nanoparticles Biosynthesized by Co-Precipitation Method
Source: Materials (Basel). 2023 Aug 3;16(15):5457. doi: 10.3390/ma16155457 (PMC10420328; doi:10.3390/ma16155457)
Supplement: Supplementary file 1 [file materials-16-05457-s001.zip › materials-2435035-supplementary.pdf]

SUPPLEMENTARY INFORMATION

FIGURES

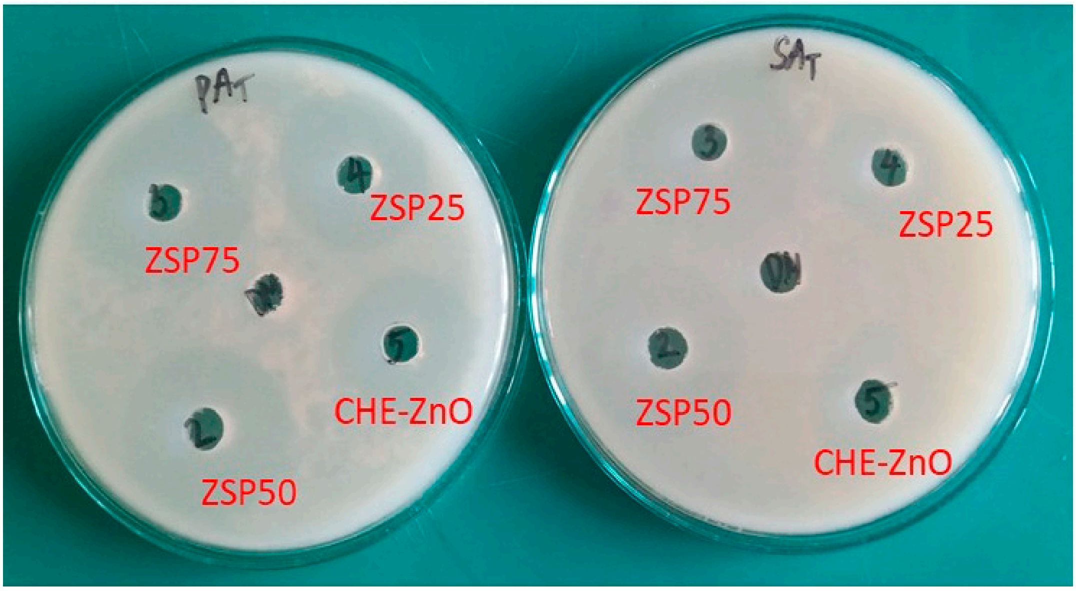

Figure S1. Antibacterial activity of CHE-ZnO, ZSP25, ZSP50 and ZSP75 sample against *P. aeruginosa* (PA$_T$) and *S. aureus* (SA$_T$)

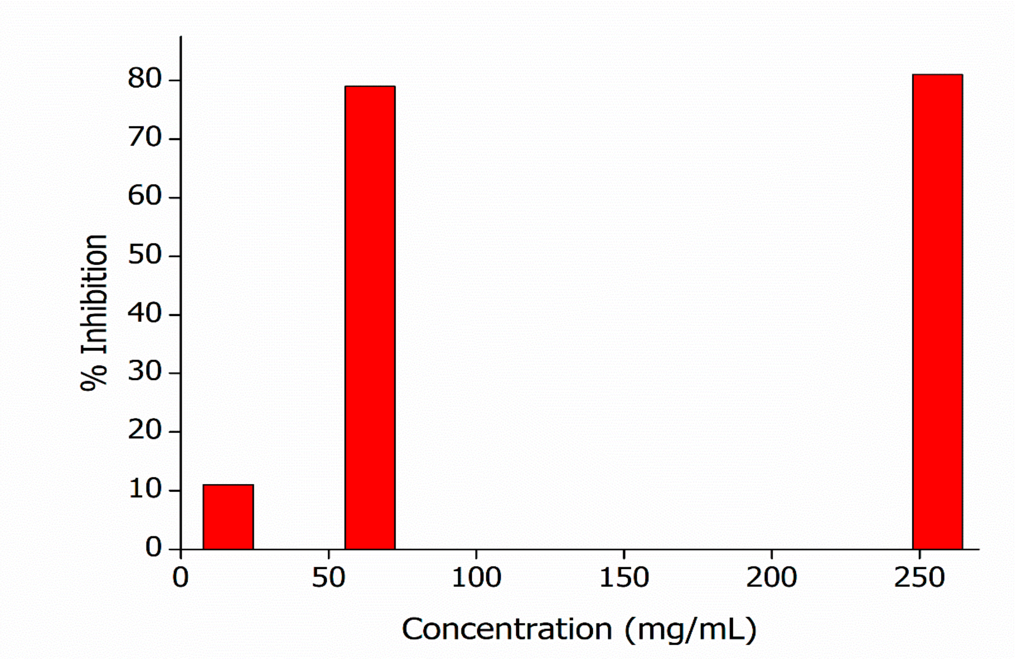

Figure S2. Anticancer activity of various concentrations of ZPS50 against KB

TABLES

Table S1. Antibacterial activity of synthesized ZnO NPs samples against *P. aeruginosa* and *S. aureus*

Bacterial strains	Diameters of the zones of inhibition for the tested bacteria (mm)							
	**DMSO**	**CHE-ZnO**	**ZPS25**	**ZPS50**	**ZPS75**	**ZSP25**	**ZSP50**	**ZSP75**
P. aeruginosa	0	25	31	**42**	37	28	31	33
S. aureus	0	22	35	**39**	36	24	30	32

Table S2. Cytotoxic activity against KB human cancer cell line of ZPS50

**No.**	**Concentration (µg/mL)**	**Inhibition (%)**
1	256	81
2	64	79
3	16	11
4	4	0
	**IC_{50}**	**43.53 ± 2.98**
Ellipticine	**IC_{50}**	**0.31±0.05**
